# An integrated analysis of second- and third-generation transcriptome sequencing technologies reveals the *DAZAP1* function in pig testis

**DOI:** 10.1590/1984-3143-AR2024-012

**Published:** 2025-06-27

**Authors:** Xia Zhang, Hailong Huo, Honglin Li, Yongqing Liu, Fujie Qiao, Changyao Li, Jinlong Huo

**Affiliations:** 1 Department of Biological and Food Engineering, Lyuliang University, Lvliang, Shanxi, China; 2 College of Animal Science and Technology, Yunnan Agricultural University, Kunming, Yunnan, China; 3 Yunnan Open University, Kunming, Yunnan, China

**Keywords:** Banna mini-pig inbred line (BMI), DAZAP1, spermatogenesis, alternative splicing isoform, transcriptional regulation

## Abstract

The quality of pig sperm is one of the crucial determinants of reproductive ability, and sperm defects can shorten the reproductive life of boars and affect the production of offspring. During transcription and translation, the *DAZAP1* gene exerts regulatory control over alternative splicing, thereby exerting influence on vital cellular processes including cell growth, development, and spermatogenesis. In this study, we employed second- and third-generation transcriptome sequencing techniques to isolate and identify the *DAZAP1* gene and its transcripts using Banna mini-pig inbred line (BMI) testicular cDNA as a template. We identified three splice variants of the *DAZAP1* gene, including ENSSSCT00000023438.4 (DAZAP1_X1), ENSSSCT00000051975.3 (DAZAP1_X2), and ENSSSCT00000074738.2 (DAZAP1_X3). Furthermore, the transcript DAZAP1_X2, was subjected to comprehensive analysis. The DAZAP1_X2 variant comprises 13 exons, with a coding sequence (CDS) length of 1254 bp (417 aa). Subsequently, enrichment analyses based on GO and KEGG pathways revealed that DAZAP1_X2 primarily participated in pathways associated with spermatogenesis, movement of the 9+2 cilium structure, germ cell development, gamete generation, and sexual reproduction. The ceRNA analysis identified three miRNAs interacting with DAZAP1_X2: ssc-miR-107, ssc-miR-127, and ssc-miR-1343, which were primarily linked to spermatogenesis. Both the testis and urethral bulb had significant levels of *DAZAP1* expression, according to multi-tissue expression analysis. Subcellular localization indicated that the DAZAP1 was mainly distributed in the cell nucleus. *DAZAP1* was critical for sperm formation and was essential for reproductive. These results shed light on the biological roles of *DAZAP1* in pigs.

## Introduction

Spermatogenesis is the procedure by which germ cells produce elongated sperm through mitosis, differentiation, and meiosis. This process involves significant chromatin condensation in the nucleus, which impacts sperm motility and fertilization (Cho et al., 2001; Hao et al., 2021; Hao et al., 2020). Spermatogenesis primarily occurs in the seminiferous tubules of the testes, where the somatic and sertoli cells are essential for the production and function of spermatozoa (Neto et al., 2016; Cheng and Mruk, 2010). After germ cell development, mature spermatids are released from the sertoli cells into the tubule lumen and travel through the rete testis and efferent ducts into the epididymis. During this passage, they undergo biochemical changes that enable motility and fertilization capability (Leblond and Clermont 1952; You et al., 2021).

The *DAZ* gene family is primarily expressed in germ cells and encodes germ cell-specific RNA binding proteins characterized by a conserved DAZ domain (Tsui et al., 2000). In the human genome, this family includes three members: *BOULE*, *DAZL*, and *DAZ* (Reijo et al., 1995; Saxena et al., 1996). DAZ-associated protein 1 (DAZAP1) was identified in RAW macrophages as an RNA-binding protein that becomes phosphorylated upon LPS stimulation (Morton et al., 2006). DAZAP1 is ubiquitously present in germ cells during late testicular development (Vera et al., 2002). It interacts with the Y-chromosome-encoded DAZ protein and the autosomal DAZ-like protein DAZL, playing an essential role in normal development and spermatogenesis(Dai et al., 2001). DAZAP1 contains two RNA binding domains (RBDs) and a proline-rich C-terminal region, which regulates the RNA life cycle from early transcription to decay (Vera et al., 2002; Smith et al., 2011). Additionally, *DAZAP1* is involved in RNA transcription, splicing, processing, transport, and localization, playing a key role in post-transcriptional regulation (Wang et al., 2021; Sasaki et al., 2018). *DAZAP1* is vital for normal growth, development, and fertility in mice, with allelic knockout leading to severe defects in spermatogenesis and cell growth, underscoring its crucial role in both germ cell and somatic cell development (Dai et al., 2001).

The Banna mini-pig inbred line (BMI), derived from the indigenous Diannan Small-ear pigs (DSE), has subjected to 44 years of highly inbred breeding using full-sibling and parent-offspring mating, representing a large mammalian model, which offers a genetically highly homozygous and well-characterized experimental animal model for biomedical research, xenotransplantation, and transgenic animal studies (Wang et al., 2023; Huo et al., 2022). However, partial infertility in some inbred boars has led to discontinuities in lineages, hindering the population expansion. Currently, the studies on *DAZAP1* are primarily focused on mice, rats, and humans. Therefore, it is crucial to investigate the molecular functions and regulatory roles of *DAZAP1* in pigs. This study aims to identify alternative splicing isoforms of *DAZAP1* using combined second- and third-generation transcriptome sequencing techniques (Hu et al., 2021), and to elucidate the specific functions and molecular mechanisms of the key transcripts regulating the expression of genes associated with pig sperm production, maturation, and fertilization at the molecular and cellular levels. Overall, this study will lay the foundation for the continuation of breeding in boars, providing fundamental data for understanding the regulatory mechanisms of spermatogenesis in BMI testes.

## Methods

### Second- and third-generation transcriptome sequencing

Testicular samples were collected from four 12-month-old BMI males, and transcriptome profiling of *DAZAP1* in the testis was conducted using a combined approach of second- and third-generation sequencing technologies. *DAZAP1* transcripts were visualized using the Sashimi plot function in IGV. The reference genome index was then constructed using STAR-2.5.2 (Dobin et al., 2013). Finally, the expression of the *DAZAP1* was visualized with the R package Gviz.

### Amplification and identification of different transcripts

The complete coding region of the *DAZAP1* mRNA sequence was obtained from the NCBI database and used as a template. Primers for gene coding region cloning were designed using Oligo7. Three primer pairs were designed based on different transcripts and labeled as F1R1 (F1: TGGGGCCAGTGACCTTCCG; R1: GCGTCCAAAGCCACTTCCG), F2R2 (F2: ACTGGAGCACCACCCAAGAGAC; R2: TGACCGCGTCCAAAGCCACT), as well as F3R3 (F3: CTTTTCCCAGTATGGCGAGGT; R3: ACTTCAGTCCAGACACCCACA). Traditional PCR was employed for amplification, followed by electrophoresis detection.

### Characterization of vital transcripts of *DAZAP1*

The nucleotide and protein sequences of *DAZAP1* obtained from sequencing were inspected and edited using the Lasergene 7.1 software package. Open reading frames (ORF) were obtained from the complete coding sequence of the gene. We conducted sequence alignment, secondary structure and tertiary structure analyses using Seqman, SPOMA (Sapay et al., 2006) and AlphaFold3 (Abramson et al., 2024). Additionally, we utilized ExPASy (Gasteiger et al., 2005) and NetPhos 3.1 (Blom et al., 2004) to analyze protein hydrophilicity and protein functional modification sites, respectively. BLAST and MegAlign were employed to analyze conserved domains and evolutionary relationships.

### Protein-protein interaction analysis

Protein-protein interaction (PPI) network was constructed using String 11.5, and functional enrichment analysis, including GO and KEGG, was performed using the clusterProfiler with a significance threshold set at *P* < 0.05. Finally, the identified proteins were correlated with gene expression data to assess expression relationships.

### Regulatory network analysis

To analyze molecular function, biological processes, and cellular components, we annotated the *DAZAP1* using the UniProt database. To identify miRNAs and lncRNAs regulating *DAZAP1*, transcriptome data were analyzed and the ceRNA transcriptional regulatory network was subsequently constructed.

### Multi-tissue expression profiling

The cDNA templates from BMI tissues, including the heart, liver, spleen, lungs, kidneys, gonads, and accessory glands, were used to assess the expression levels of the *DAZAP1* gene across various tissues. The qPCR primers (F4: CCGAAGGAAGGATGGCAGAAA; R4: CACTTCTGTGACCACTCCAAAT) were designed at the exon-exon junction using Seqman and Oligo7 software. The *GAPDH* (F5: CCTTCATTGACCTCCACTACATGGT; R5: CCACAACATACGTAGCACCAGCATC) was used as a housekeeping gene. Data analysis followed the relative quantification 2^-ΔΔct method method (Rao et al., 2013).

### Subcellular localization

Transfection was conducted when Swine Testis (ST) cell confluence reached 70-80% in each well. The cells were then incubated with 5% CO_2_. Successful transfection was confirmed by observing green fluorescent protein expression under an inverted fluorescence microscope. Mitochondria were stained with Mito Tracker, and nuclei with Hoechst33342 to determine the intracellular localization of the target protein.

## Results

### Different alternative splicing isoforms of *DAZAP1*

**Figure 1:**
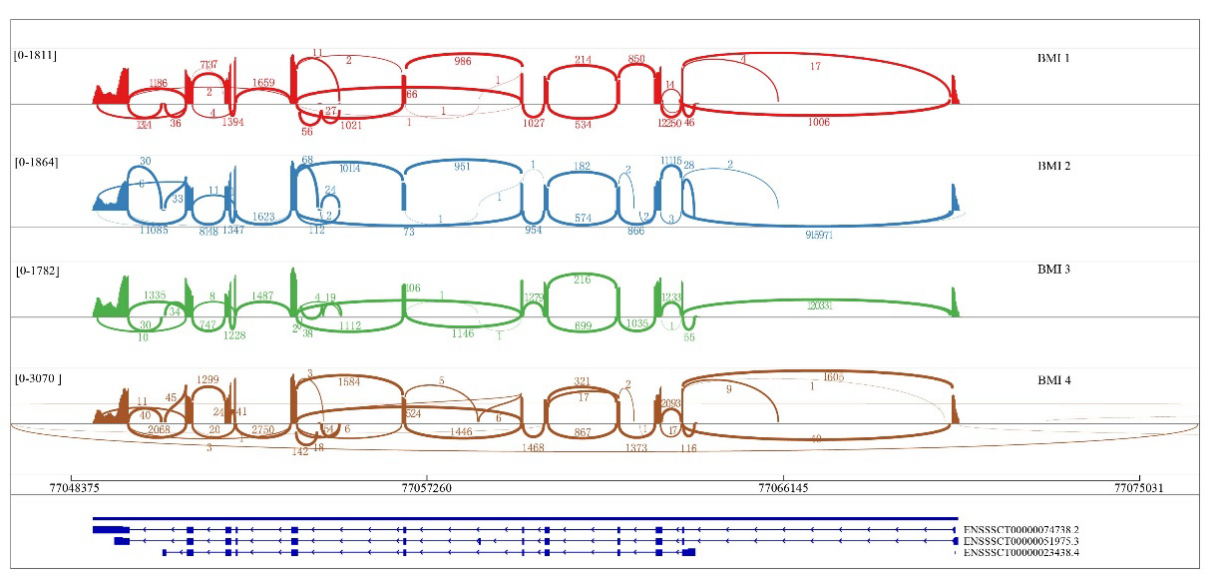
Gene structure of alternative splicing variants of porcine *DAZAP1* gene. The four different colors in the figure represent four distinct samples. The numbers above the arcs indicate expression levels, with higher numbers representing higher expression and lower numbers representing lower expression. The three tracks below represent three different transcripts (ENSSSCT00000023438.4, ENSSSCT00000051975.3, and ENSSSCT00000074738.2).

The second- and third-generation sequencing results showed that there were three alternative splicing isoforms of *DAZAP1* in the BMI pig testes, namely ENSSSCT00000023438.4, ENSSSCT00000051975.3, and ENSSSCT00000074738.2, which were named here DAZAP1_X1, DAZAP1_X2, and DAZAP1_X3, respectively (Figure 1). The results showed that our four samples matched the same transcript, which had the most exons and was consistent with the annotated transcript. DAZAP1_X1 variant consists of 11 exons, with a CDS length of 1197 bp (398 aa), including 23.31% A, 26.98% G, 31.00% C, and 18.71% T. The DAZAP1_X2 variant consists of 13 exons, with a CDS length of 1254 bp (417 aa), including 23.37% A, 17.38% T, 27.67% G, and 31.58% C. The DAZAP1_X3 variant comprises 12 exons, with a CDS length of 1221 bp (406 aa), including 23.26% A, 16.79% T, 28.17% G, and 31.78% C. Of note, DAZAP1_X2 had the longest CDS and the most exons, making it the major transcript.

### Sequence verification and identification of different *DAZAP1* transcripts

**Figure 2:**
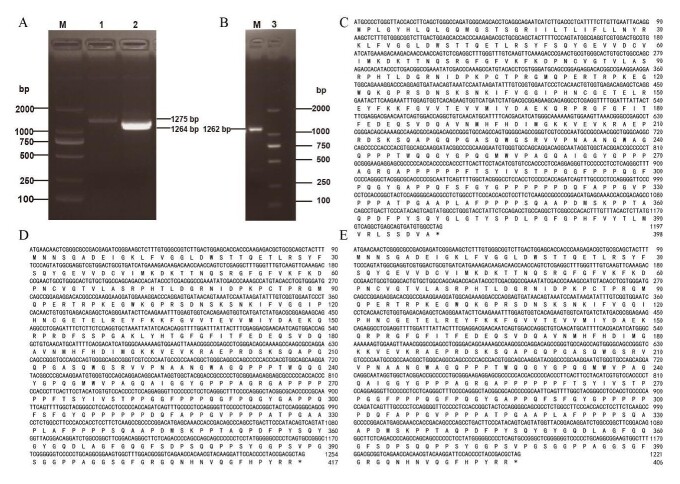
Identification of distinct transcripts of *DAZAP1*. (A-B) CDS sequence amplification of different transcripts of *DAZAP1*, M: DL2000 Marker; (C) Nucleic acid protein sequence of DAZAP1_X1; (D) Nucleic acid protein sequence of DAZAP1_X2; (E) Nucleic acid protein sequence of DAZAP1_X3. ATG: start codon, “*”: stop codon.

After ORF prediction and sequence alignment, it was determined that the three amplified sequences correspond to the three transcripts of the *DAZAP1* gene. Using cDNA from BMI testicular tissues as a template, three pairs of specific primers were designed to amplify three different splicing isoforms of the *DAZAP1* gene. Agarose gel electrophoresis confirmed that the amplified fragments matched the expected results, with lengths of 1275 bp, 1264 bp, and 1262 bp, respectively (Figure 2A-2B). PCR results indicated that the amplified fragments from the three primer pairs included the complete coding sequences (CDS) and the portion of the 5' and 3' untranslated regions (UTRs) (Figure 2C-2E). Consistent with the transcriptome data, DAZAP1_X2 emerged as the predominant transcript.

### Sequence feature analysis and function prediction of DAZAP1_X2

**Figure 3:**
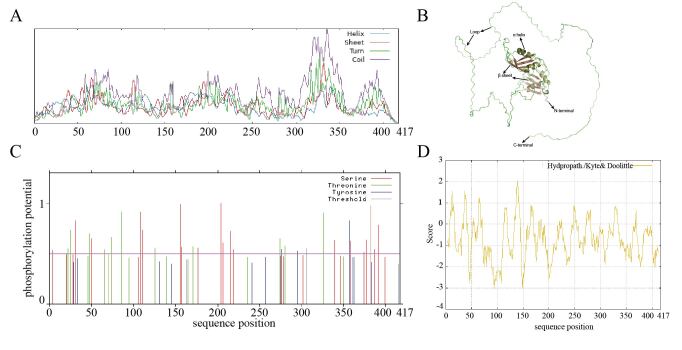
Structure of DAZAP1 protein. (A) Secondary structure; (B) Tertiary structure; (C) Phosphorylation site; (D) Hydrophobicity.

The molecular weight of pig DAZAP1_X2 protein is 44.50 kDa, with a molecular formula of C_2001_H_2967_N_551_O_586_S_12_, and an isoelectric point of 8.71. The secondary structure of the DAZAP1 protein, comprises 12.23% α-helix (51 aa), 5.52% β-turn (23 aa), 12.95% extended strand (54 aa), and 69.30% random coil (289 aa), respectively (Figure 3A). The tertiary structure of DAZAP1 protein is consistent with the secondary structure analysis results (Figure 3B). DAZAP1 contains phosphorylation sites for serine, threonine, and tyrosine residues (Figure 3C). The N-terminus of the DAZAP1 is hydrophobic, while the C-terminus is hydrophilic, with the maximum hydrophobicity value being 2.067 at the 139th amino acid position, and the minimum hydrophobicity value being -3.033 at the 94th amino acid position (Figure 3D).

### Conservation and evolutionary relationship of DAZAP1_X2 in multiple species

**Figure 4:**
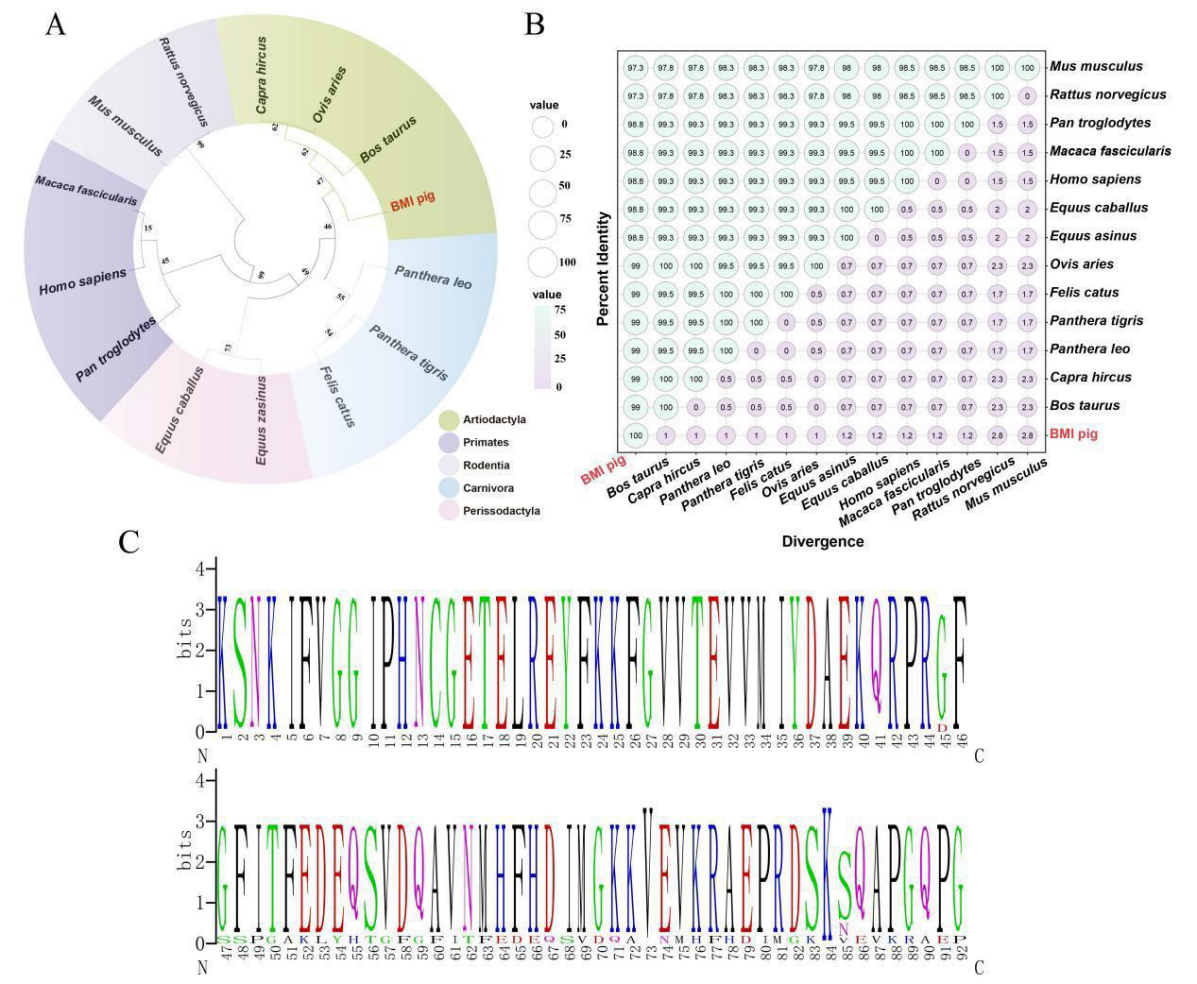
Comparison of the phylogenetic tree, similarity, and conserved domains of the amino acid sequences of DAZAP1. (A) Phylogenetic tree; (B) Similarity; (C) Conserved domain.

Comparison of amino acid sequences across 13 species classified pigs, along with goats, sheep, and cattle, as artiodactyls; cats, lions, and tigers as carnivores; mice and rats as rodents; humans, gorillas, and cynomolgus monkeys as primates; and horses and donkeys as ungulates (Figure 4A). The similarity between the BMI pig and the sequences from cattle (*Bos taurus* XP_024850355), goat (*Capra hircus* XP_017905929), donkey (*Equus asinus* XP_044609611), horse (*Equus caballus* XP_023502104), cat (*Felis catus* XP_023098767), human (*Homo sapiens* EAW69502), cynomolgus monkeys (*Macaca fascicularis* XP_045235029), mouse (*Mus musculus* NP_573451), lion (*Panthera leo* XP_042783741), tiger (*Panthera tigris* XP_042833498), chimpanzee (*Pan troglodytes* XP_054529711), and rat (*Rattus norvegicus* NP_001020913) was above 90% (Figure 4B), indicating a relatively conserved sequence. All branches conformed to the systematic classification standards. Analysis of 14 conserved domains of mammalian DAZAP1 using Weblogo revealed a high level of conservation across species (Figure 4C). In summary, the *DAZAP1* gene in BMI pigs exhibited significant conservation and sequence homology with other mammals throughout evolution.

### Protein-protein interactions of DAZAP1_X2

**Figure 5:**
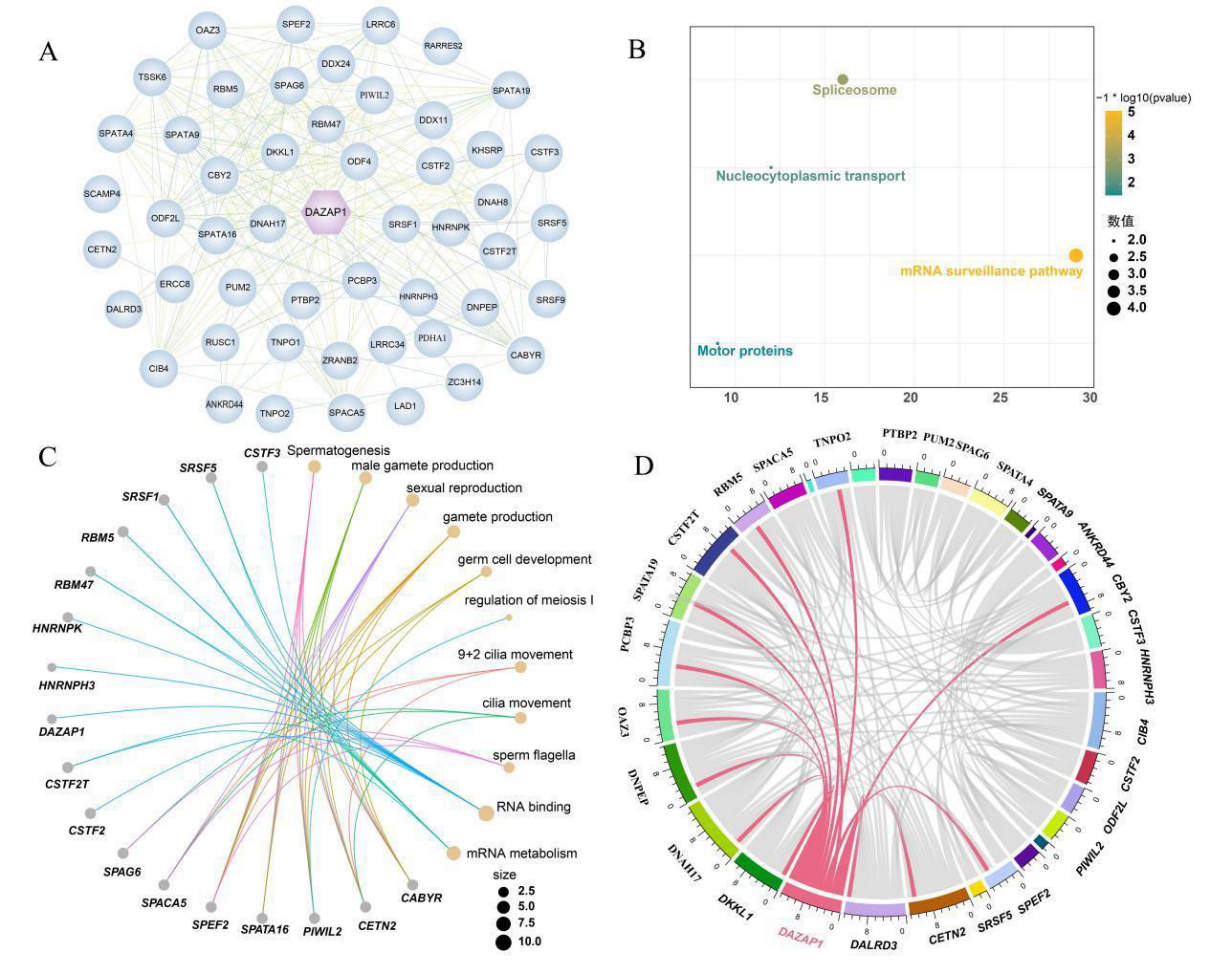
Protein interaction analysis. (A) protein- protein interacting protein network; (B) KEGG enrichment analysis diagram of interacting proteins; (C) GO enrichment analysis diagram of interacting proteins; (D) Correlation chord diagram.

The pig DAZAP1 protein had interactions with various proteins including dickkopf-like acrosomal protein 1 (DKKL1), the outer dense fiber of sperm tails 4 (ODF4), RUN and SH3 domain-containing 1 (RUSC1), DEAD-box helicase 24 (DDX24), zinc finger CCCH-type containing 14 (ZC3H14), spermatogenesis associated 9 (SPATA9), cleavage stimulation factor subunit 2 (CSTF2), serine and arginine-rich splicing factor 1 (SRSF1), RNA binding motif protein 5 (RBM5), sperm acrosome-associated 5 (SPACA5), sperm as-sociated antigen 6 (SPAG6) and so on (Figure 5A). These proteins focused on various biological processes including protein secretion, mRNA translation, spermatogenesis, and cell growth and division. KEGG pathways enrichment analysis identified four significant pathways (*P* < 0.05) for these proteins, with notable enrichment in spliceosomes, nuclear transport, mRNA surveillance pathways, and monitoring proteins (Figure 5B). Moreover, GO enrichment analysis revealed that these proteins were predominantly associated with spermatogenesis, male gamete generation, sexual reproduction, gamete generation, germ cell development, regulation of meiotic nuclear division I, 9+2 flagellar movement, sperm flagellum, RNA binding, and mRNA metabolic processes (Figure 5C). Correlation analysis using transcriptome sequencing data revealed a significant correlation (*P* < 0.05) between DAZAP1 and DKKL1, DNAH17, DNPEP, OAZ3, PCBP3, SPAZA19, CSTF2T, RBM5, TNPO2, CBY2, SRSF5, CETN (Figure 5D).

### Functional annotation and ceRNA regulatory network of DAZAP1_X2

**Figure 6:**
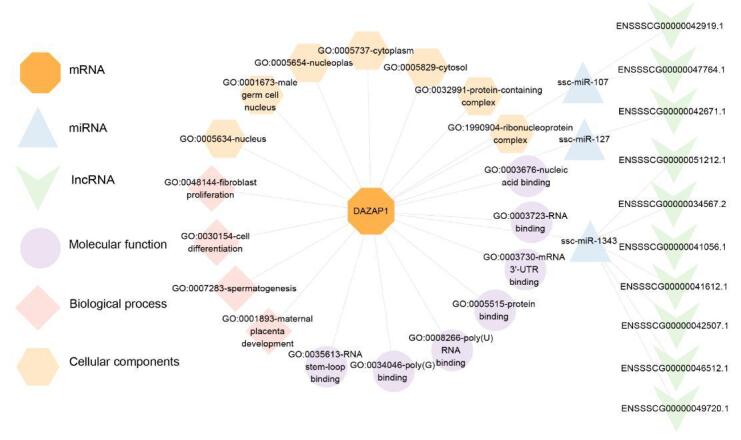
Functional annotation and ceRNA regulatory network of *DAZAP1.*

Functional annotation of *DAZAP1* identified 18 Gene Ontology (GO) terms, including biological processes such as fibroblast proliferation, cell differentiation, spermatogenesis, and maternal placenta development. Cell components included the nucleoplasm, ribonucleoprotein complex, protein domain complex, cytoplasm, cell nucleus, and male germ cell nucleus. Molecular functions encompassed RNA stem-loop binding, poly(G) binding, poly(U) RNA binding, protein binding, mRNA 3' UTR binding, RNA binding, and nucleic acid binding. ceRNA regulatory network showed that *DAZAP1* was targeted regulation by ssc-miR-107, ssc-miR-127, and ssc-miR-1343. Futhermore, two lncRNAs (ENSSSCG00000042919.1 and ENSSSCG00000047764.1) competitively bind to ssc-miR-107 targeting *DAZAP1*, while one lncRNA (ENSSSCG00000042671.1) competitively binds to ssc-miR-127, and seven lncRNAs (ENSSSCG00000051212.1, ENSSSCG00000034567.2, ENSSSCG00000041056.1, ENSSSCG00000041612.1, ENSSSCG00000042507.1, ENSSSCG00000046512.1, ENSSSCG00000049720.1) competitively bind to ssc-miR-1343, affecting *DAZAP1* regulation (Figure 6).

### Expression profiles in multiple tissues analysis of *DAZAP1_X2*

**Figure 7:**
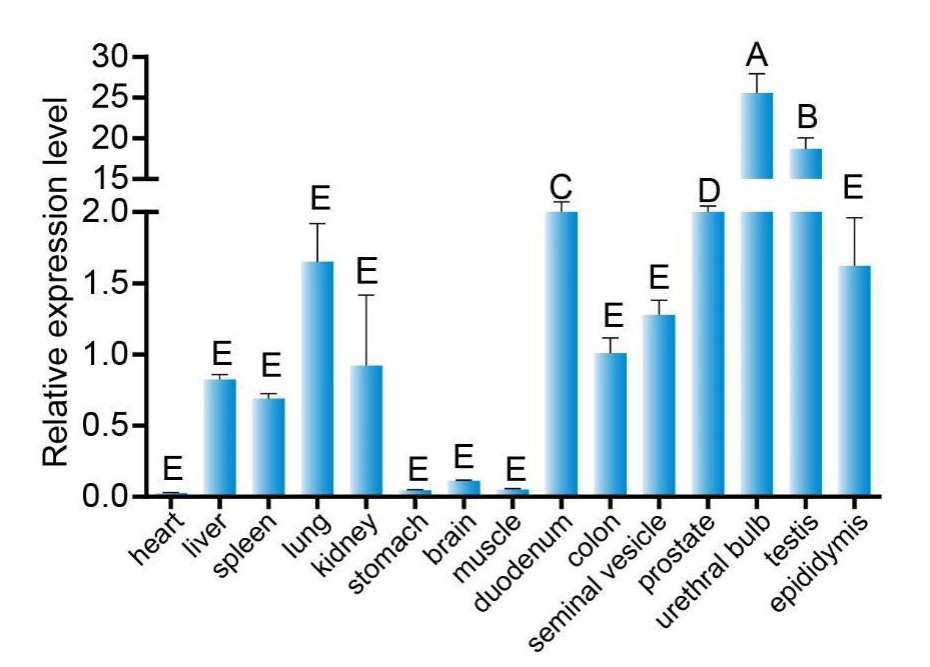
Multi-tissue expression profile of BMI *DAZAP1* gene. Different capital letters on the bars indicate significant differences (*P*<0.01).

To investigate the expression of the *DAZAP1_X2* across various tissues, relative quantification using qPCR was performed with *GAPDH* as the housekeeping gene. The 2^-ΔΔCt^ method was utilized for relative quantification. The results revealed varying degrees of expression across the 15 tissues examined. DAZAP1 mRNA showed relatively higher expression in glandular organs such as the prostate, urethral bulb, testis, and epididymis. Moderate expression was observed in the duodenum, colon, heart, spleen, lungs, and kidneys, while relatively lower expression was detected in other tissues (Figure 7).

### Subcellular localization analysis of DAZAP1_X2

**Figure 8:**
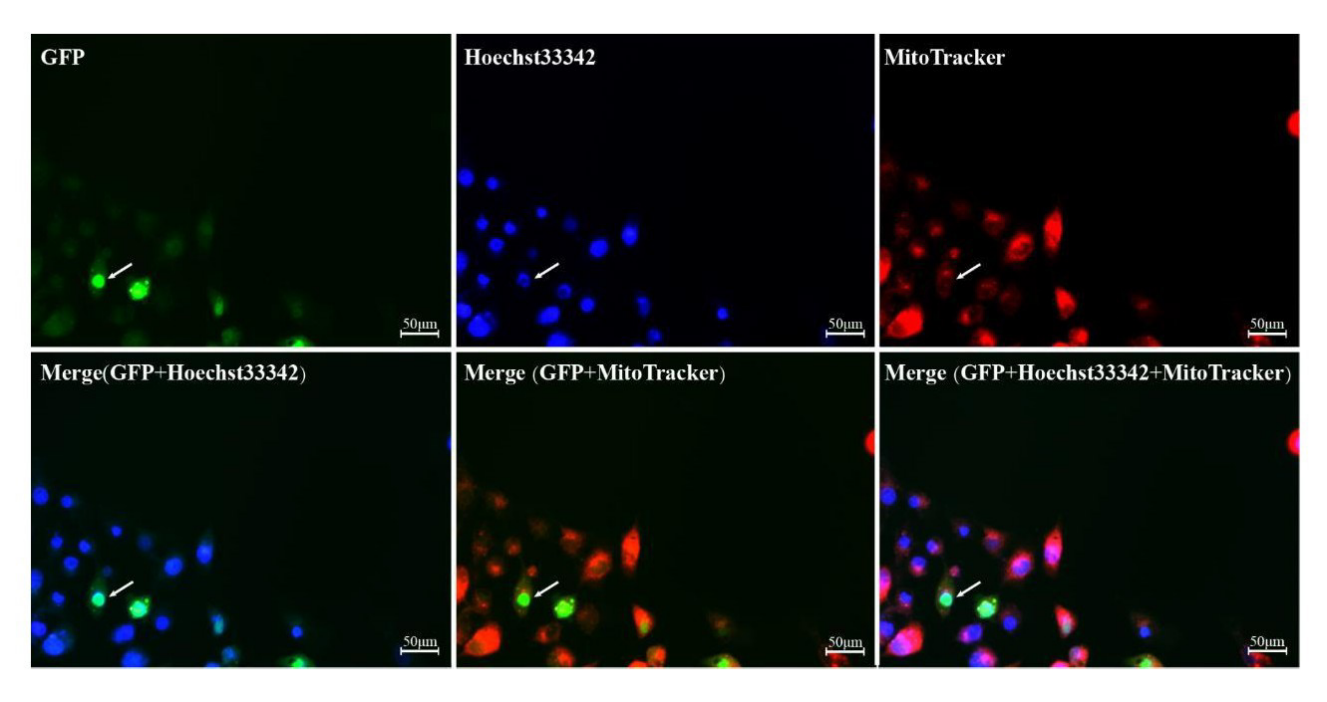
Subcellular localization of DAZAP1 protein.

The Swine Testis (ST) cells were transfected with pEGFP-C1-*DAZAP1* and pEGFP-C1 control plasmids. After 24 hours of expression, green fluorescence was observed. Subsequently, the cells were processed with MitoTracker Red (mitochondrial staining) and Hoechst33342 (nuclear staining), followed by observation under an inverted fluorescence microscope. In ST cells transfected with the pEGFP-C1-*DAZAP1* plasmid, green fluorescence was predominantly localized in the cell nucleus (Figure 8). To further analyze the DAZAP1 protein localization, we merged the green, red, and blue fluorescence in the same field of view, suggesting that DAZAP1 is mainly located in the cell nucleus.

## Discussion

Alternative splicing is the process by which exons of primary gene transcripts (pre-mRNA) are spliced in different ways, resulting in the production of mRNA and protein variants with different structures and functions (Blencowe, 2006). Proteins translated from selectively spliced mRNAs typically exhibit differences in amino acid sequences and biological functions. Selective splicing coordinates physiologically meaningful changes in the expression of protein isoforms, representing a key mechanism in the generation of complex proteomes in multicellular organisms (Lee and Rio, 2015). In this study, the testicular tissue of BMI pigs was sequenced using second- and third-generation transcriptome sequencing technologies. The coding sequences of three splice isoforms of the *DAZAP1* gene, namely DAZAP1_X1, DAZAP1_X2, and DAZAP1_X3, were obtained using molecular cloning techniques. Among these isoforms, DAZAP1_X2 is the most complete, while DAZAP1_X1 lacks the 7th exon and the 5' UTR region, and DAZAP1_X3 lacks the 7th exon, indicating an exon skipping splicing pattern. Evolutionary analysis indicated that BMI DAZAP1 shared a close evolutionary relationship with cattle, sheep, and goats. Its similarity to mammals such as rats, mice, horses, and donkeys exceeds 90%, suggesting a high conservation and sequence homology of BMI DAZAP1 with other mammals in evolution. Protein domain analysis revealed that the DAZAP1 protein contained an RNA recognition motif (RBM) domain of serine/arginine protein kinase expressed exclusively in the nuclei of male germ cells.

Protein interaction network analysis revealed that DAZAP1 interacted with several proteins, including DKKL1, ODF4, DDX24, ZC3H14, SPATA9, SPACA5, and SPAG6. DKKL1 has been independently identified as a distant homolog of the Dickkopf (Dkk) protein family, known to modulate wnt/β-catenin signaling. It is specifically expressed in testicular tissue, primarily localized in spermatogonia and round spermatids. In patients with azoospermia, the expression of *DKKL1* in testicular tissue is significantly reduced or absent compared to that in normal testicular tissue (Yan et al., 2012). The proper shape and movement of sperm tails are crucial for fertilization. Outer dense fiber 4 (ODF4) is specifically expressed in the testes. Knockout of *ODF4* in male mice results in curved and abnormally motile sperm tails, leading to male infertility (Ito et al., 2023). The Glu-Ala-Asp motif, characteristic of the DEAD-box family, represents an ATPase-active RNA helicase. *DDX24* plays a significant role in various biological processes, including embryonic development, spermato-genesis, cell growth, and tumorigenesis (Chen et al., 2022). Expression of *SPATA9* varies in patients with spermatogenic arrest, with not being expressed in patients arrested at the spermatogonia and primary spermatocyte stages (Cheng et al., 2003). Circular RNAs (circRNAs) are natural byproducts of eukaryotic transcription and RNA processing and have emerged as key regulatory fac-tors in physiology and human diseases. *ZC3H14* binds to the spliceosome, interacting with the 3' and 5' exons-introns boundaries of circular exons, and forms dimers to promote circRNA biogenesis. Knockout mice lacking *ZC3H14* exhibit disrupted spermatogenesis and reduced levels of testicular circRNAs (Rha et al., 2017). *SPACA5*, also known as lysozyme-like pro-tein 5 (LYZL5), encods a putative protein of 159 amino acids, and is located on the X chromosome at p11.23. The SPACA5 protein is observed only in the elongated spermatids of male spermatogenesis (Chen et al., 2006). The *SPAG6* encodes an axonemal protein maintaining the structure and function of the axoneme (Neilson et al., 1999). Variants of *SPAG6* are associated with multi-systemic disorders involving cilia and flagella (Wu et al., 2020). The biallelic mutations in *SPAG6* are associated with primary ciliary dyskinesia (PCD) (Neilson et al., 1999). Moreover, we found that these proteins were primarily involved in spermatogenesis, male gamete production, sexual reproduction, 9+2 flagellar movement, and meiosis. The discovery of these proteins potentially interacting with DAZAP1 provided new insights into understanding the function of this gene.

Competing endogenous RNAs (ceRNAs) are a class of short non-coding RNAs that regulate gene expression post-transcriptionally by inhibiting translation or promoting mRNA degradation (Cai et al., 2009). Among them, long non-coding RNAs (lncRNAs), mRNAs, and other RNAs function as natural miRNA sponges, inhibiting target mRNAs. LncRNAs regulate protein levels and influence cellular processes by competing with miRNAs (Poliseno et al., 2010). Functional annotation of DAZAP1 in this study revealed its involvement primarily in spermatogenesis and cell differentiation processes. Target gene prediction suggested that *DAZAP1* was regulated by three miRNAs: ssc-miR-107, ssc-miR-127, and ssc-miR-1343. Unlike most miRNAs that target complementary sequences in the 3'UTR region, certain members of the miR-107 family uniquely target the coding sequence of mRNA, thereby inhibiting translation without interference. MicroRNAs (miRNAs) play a crucial role in adipogenesis, with miR-127 promoting proliferation by enhancing the cell cycle and inhibiting differentiation, thus reducing lipid accumulation (Gao et al., 2019). miR-1343 increases the expression of pluripotency genes by inhibiting OTX2, thereby maintaining the pluripotency of porcine embryonic stem cells (Xie et al., 2019). Additionally, miR-1343 overexpressed in capacitated sperm which undergo critical physiological changes necessary for fertilization, suggesting that miR-1343 can serve as a therapeutic target to alleviate the impact of infertility (Sahoo et al., 2021). Therefore, the predicted association of miR-1343 with male infertility underscored its potential as a therapeutic target for early diagnosis and prognosis of male infertility.

DAZAP1 is a ubiquitously expressed hnRNP (heterogeneous nuclear ribonucleoprotein) involved in RNA transcription, splicing, and transport (Hsu et al., 2008; Yang et al., 2009). The DAZAP1 is expressed in various human and mouse tissues (Reijo et al., 2000), with high expression observed in the testes, and lower in other tissues (Dai et al., 2001). In BMI pigs, *DAZAP1* was highly expressed in the testes and seminal vesicles, while its expression was relatively low in other tissues, especially in the heart, stomach, brain, and muscles. In male reproduction, accessory glands including the prostate, seminal vesicles, and urethral bulb (Gilany et al., 2015). Seminal plasma is rich in tissue-specific proteins and peptides that bind directly to sperm (Manjunath et al., 2007). The transport of large molecules, such as proteins or RNA, between the nucleus and cytoplasm occurs through the nuclear pore complex (NPC). The NPC is a selective and bidirectional transport channel for various cargo molecules, recognized by nuclear transport proteins via nuclear export signals (Kim et al., 2017). During spermatogenesis, DAZAP1 exhibits dynamic subcellular localization primarily in the nuclei of late spermatocytes and round spermatids (Lin and Yen 2006). In porcine testicular cells, DAZAP1 is primarily localized in the nuclear, with minimal presence in the cytoplasm. In summary, *DAZAP1* deletion or abnormalities can lead to spermatogenesis defects, highlighting its crucial role in spermatogenesis.

## Conclusion

The integration of second- and third-generation transcriptome sequencing offered a detailed analysis of *DAZAP1*’s transcriptional regulation, identifying three distinct transcripts. Additionally, 50 interacting proteins were identified, primarily focusing on protein secretion, mRNA translation, spermatogenesis, and cell growth and division. Significant associations were found between DAZAP1 and genes such as *DKKL1*, *DNAH17*, *DNPEP*, *OAZ3*, *PCBP3*, *SPAZA19*, *CSTF2T*, *RBM5*, *TNPO2*, *CBY2*, *SRSF5*, and *CETN*. *DAZAP1* was associated with 18 GOs and was targeted by three miRNAs. *DAZAP1* was highly expressed in the gonads and accessory glands. These results enhance our insight into *DAZAP1*, contributing to the clarification of its role and mechanism in spermatogenesis within BMI pigs.
